# Intraoperative control of air leak using a sutureless free pericardial fat pad covering method in lung cancer resection

**DOI:** 10.1111/1759-7714.15065

**Published:** 2023-08-10

**Authors:** Sachie Koike, Nobutaka Kobayashi, Masahisa Miyazawa

**Affiliations:** ^1^ Department of Thoracic Surgery Japanese Red Cross Society Nagano Hospital Nagano Japan; ^2^ Division of General Thoracic Surgery Department of Surgery, Shinshu University School of Medicine Matsumoto Nagano Japan

**Keywords:** free pericardial fat pad, intraoperative air leak control, without suture

## Abstract

Here, we introduce a new method for intraoperative control of air leak using a free pericardial fat pad covering to lung damage with sutureless fixation. We covered the damaged lung tissue with a free pericardial fat pad with a polyglycol acid sheet and fibrin glue fixation. This method provides a good air leak controlling effect with the use of a free pericardial fat pad and relatively short operative time with sutureless fixation.

## Graphical abstract

We introduce a new method for the control of an intraoperative air leak without suturing. In our method we covered lung parenchymal damage with free pericardial fat pads repaired with fibrin glue and a polyglycol acid sheet (A–E). This study reveals the efficacy and efficiency of our method.

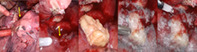

## INTRODUCTION

Air leak or prolonged air leak (PAL) after lung resection reportedly increases the risk of morbidity and mortality.^1^, ^2^, ^3^, ^4^, ^5^, ^6^ Therefore, prevention of an air leak is a significant issue.

Damage of lung parenchyma during surgery represents the most common cause of air leak after pulmonary resection,^1^ and intraoperative damage repairing techniques are important to reduce postoperative air leak. A variety of techniques have been adopted; however, there are several problems. First, lung suture techniques are difficult if there is damage near the hilum or damage to a fragile lung (e.g., chronic obstructive pulmonary disease [COPD], interstitial pneumonia [IP]). Second, it is difficult to cover complexed shaped damage with the sheet‐type sealants. Third, suturing techniques require technical skill and longer operative time especially in video‐assisted thoracoscopic surgery (VATS). To overcome these problems, we have introduced a new method which uses free pericardial fat pads (FPFP) to cover damage to the lung parenchyma without suturing (sutureless fat pad method: SFP method). The purpose of this study was to investigate the clinical outcomes of patients who were treated with this new method.

## METHODS

### Patients

This study was approved by the ethics review board of Japanese Red Cross Society Nagano hospital, Nagano, Japan (approval no.: R‐099), and the requirement for informed patient consent was waived.

In total, 78 patients underwent anatomic lung resection for the treatment of primary lung cancer at Japanese Red Cross Society Nagano hospital between February 2022 and March 2023. We divided these patients into four groups based on the type of intraoperative lung parenchymal damage (also shown in Figure S1): Type 1, incomplete staple line (intraoperative air leak from staple line); Type 2, delamination; and Type 3, laceration.

We selected 22 type 2 or 3 patients in this study as they were supposed to be at a greater risk of PAL. All type 2 and 3 patients had an air leak in the intraoperative water sealing test. To maintain uniformity, one surgeon (S.K.) operated on all patients enrolled in this study, and patients that were operated on by other surgeons were excluded (Figure S2).

### Techniques and surgical procedure

**FIGURE 1:**
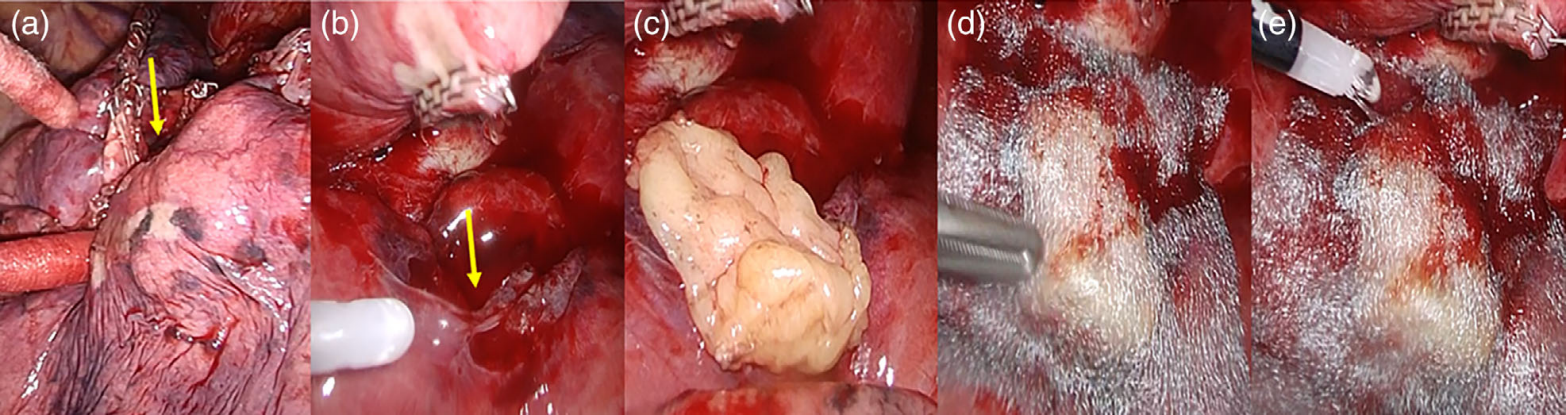
Procedure for sutureless fat pad method. (a) The lung delamination (arrow) was found near the hilum. The procedure was applied to exclude the lung. (b) Fibrin glue (arrow) was applied to the damaged area to fix the FPFP (c) The harvested FPFP was fixed to the damaged area. To fix FPFP thickly to the damaged area, We attached without pleura side of FPFP. (d) Polyglycol acid (PGA) sheet was used to cover the FPFP and the damaged lung parenchyma. (e) Fibrin glue was again applied to strengthen the fixation. FPFP, free pericardial fat pad.

After lung resection was completed, the damage of the lung parenchyma was assessed as previously described (Type 0, no damage; Type 1, incomplete staple line; Type 2, delamination; Type 3, laceration). When the damage was type 2 or 3, which is supposed to cause a greater risk of air leak, a sufficient amount of FPFP was harvested to cover the damage using LigaSure Maryland (Medtronic, Covidien Products). The harvested FPFP was fixed to the damaged area by applying fibrin‐glue (Bolheal, KM Biologics Co., Ltd.) (Figure 1a–c). The FPFP was attached without pleura side of FPFP, because it is easier to thickly fix to the damaged area. Then, polyglycol acid (PGA) sheet (Neoveil, Gunze Ltd.) was used to cover the FPFP and the damaged lung parenchyma (Figure 1d). After that, we applied fibrin glue again to strength the fixation (Figure 1e). All patients had one chest tube placed apically.

### Postoperative management

Chest tubes were placed postoperatively under negative pressure suction of 10 cmH_2_O until the patients were moved to the intensive care unit (ICU) from the operative room. After the patient was admitted to ICU the suction pressure was reduced to water seal. The chest tubes were removed on the next day following the air leak disappeared date with previous roentgenogram check.

### Assessment

Data were collected on age, gender, smoking habits, history of COPD/IP/combined fibrosis and emphysema (CPFE), type of surgery, pulmonary pathology, time for our method (time for FPFP harvesting + FPFP fixing), and duration of air leaks. Postoperative CT was checked to evaluate the residual FPFP and its position. The postoperative CT includes both CT scans performed during postoperative hospitalization and those performed in the outpatient clinic.

## RESULTS

**FIGURE 2:**
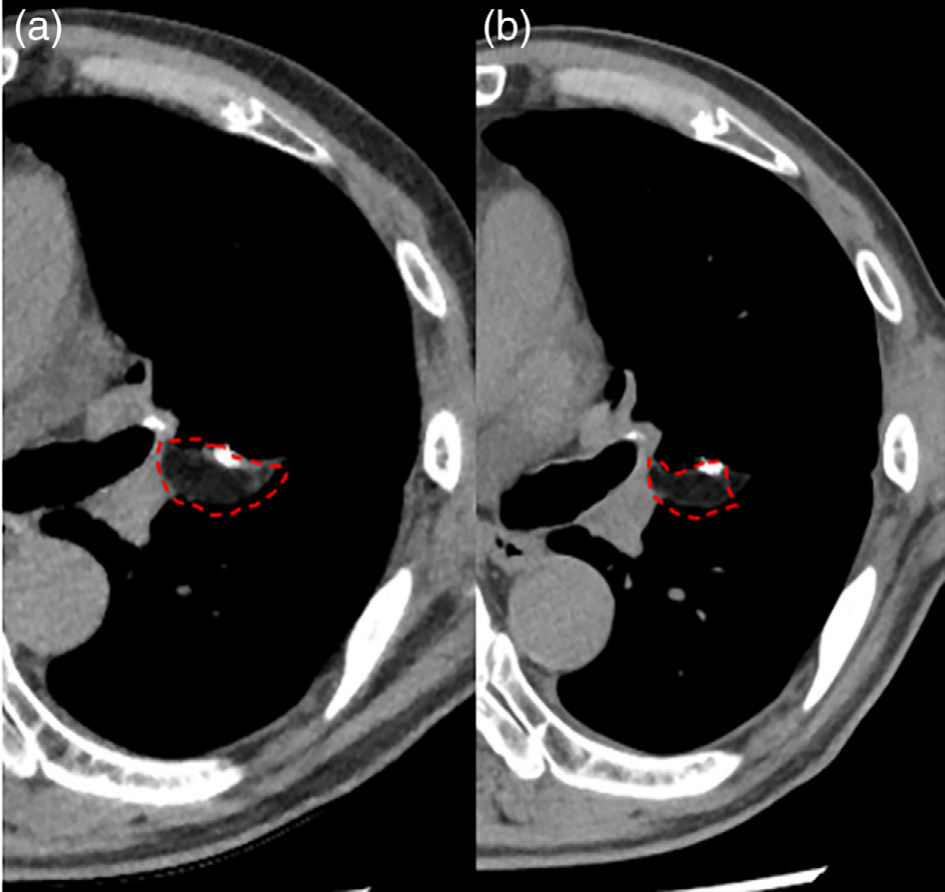
Images of the residual FPFP. (a) The residual FPFP (dotted line) of one of the patients was found on computed tomography (CT) scan undertaken 6 months after surgery. (b) The FPFP (dotted line) still remained on postoperative CT that was performed 1 year after surgery although it was slightly reduced. FPFP, free pericardial fat pad.

The characteristics of 22 patients and results of our FPFP damage covered without the suturing method are demonstrated in Table 1. A total of 15 (68.2%) patients had smoking habits, and five (22.3%) patients were diagnosed with COPD. The number of IP or CPFE patients was one in each. Surgical procedure was segmentectomy in 14 (63.6%) patients and lobectomy in eight (36.4%) patients, and the majority of the patients had undergone segmentectomy. Most of the patients (*n* = 16, 72.7%) had undergone VATS. The time for method was checked in 16 of 22 patients (VATS *n* = 15, thoracotomy *n* = 1). The median time was 169 s (25–75 percentiles, 141–262). The median air leak duration was one day (25–75 percentiles, 0–2). PAL occurred in only one patient who had CPFE. Duration of hospital stay was 5 days (25–75 percentiles, 4–8). Postoperative CT was checked in 13 patients (within 3 months *n* = 4, 6 months *n* = 8, 1 year *n* = 3 [two times evaluation *n* = 2]). A total of 10 of 13 patients (76.9%) had residual FPFP (Figure 2a, b). All of the residual FPFPs remained in the same position where they had been placed during surgery.

**TABLE 1:** **Patient characteristics and results of** sutureless fat pad method.

No. of patients	22
Gender	
Male	14
Female	8
Age	73 (69–76)
Smoking	15 (68.2)
COPD	5 (22.3)
IP	1 (4.5)
CPFE	1 (4.5)
Operative procedure	
Segmentectomy	14 (63.6)
Lobectomy	8 (36.4)
VATS/thoracotomy	
VATS	16 (72.7)
Thoracotomy	6 (27.3)
Type of lung damage	
Delamination	15 (68.2)
Laceration	7 (31.8)
Time for FPFP cover without suture^a^ (s)	169 (141–262)^a^
Air leak duration (days)	1 (0–2)
PAL	1 (4.5)
Duration of hospital stay (days)	5 (4–8)
Postoperative residual FPFP^b^	10/13 (76.9)^b^

*Note*: Categorical data are shown as no. (%) and continuous data as median, range, 25%–75% interquartilerange.

Abbreviations: COPD, chronic obstructive pulmonary disease; CPFE, combined pulmonary fibrosis and emphysema; FPFP, free pericardial fat pad; IP, interstitial pneumonia; PAL, prolonged air leak; VATS, video‐assisted thoracoscopic surgery.

^a^ Time checked in 16 of 22 patients (VATS *n* = 15, thoracotomy *n* = 1).

^b^ The postoperative CT was evaluated in 13 patients.

## DISCUSSION

A range of methods for the control of intraoperative air leaks have been previously reported, such as suturing, stapling, and covering with sheet type sealant products (oxidized regenerated cellulose [ORC] mesh, PGA sheet, or collagen sponge).^7^, ^8^, ^9^ There are problems associated with these methods which include: (1) difficulty in suturing or stapling damaged lung near the hilum, or a fragile lung (e.g., COPD, IP/CPFE). (2) It is difficult for sheet type sealant products to cover the complex shaped surface of the damaged surface of the lung. (3) The suturing method is lengthy and is considered an advanced technique, especially in VATS.

Our SFP methods overcame all of the problems described above. In our study, the median air leak duration was one day (25–75 percentiles, 0–2) and PAL occurred in only one CPFE patient. Also, the median time for our method was 169 s (25–75 percentiles, 141–262), which is relatively short. The results suggest that our lung parenchymal damage repairing method is effective and efficient.

Similar to our method, there have been some reports which have noted the efficacy of using isolated fat as a sealant. These reports have also revealed the greater effect of fat sealant than the PGA sheet + fibrin glue methods.^10^, ^11^, ^12^ Shintani et al. reported using the subcutaneous fat pad method.^10^ Ikeda et al. and Kameyama et al. reported using the FPFP method.^11^, ^12^ However, all these methods sutured fat materials to the lung damaged area, and it is necessary to deal with the problems which might arise as described in (1) and (3) above. To reveal the superiority of our method to the fat suturing methods in (3), we retrospectively investigated the time for FPFP suturing (*n* = 2, VATS) and lung suturing (*n* = 5, VATS) that had taken place before this study started (June 2020 to December 2021) as a reference data and compared this to the time for our method. The median time for our method was 167 s (25–75 percentiles, 137–279) as mentioned above; FPFP suturing was 812 (693–932), and lung suturing 421 (280–491) (Table S1). The time for our method was obviously shorter than using suturing methods.

Similar to our method, Yoshimura et al. reported using the pedicled pericardial fat pad method with fibrin glue fixation to the lung.^13^ Harvesting pedicled pericardial fat pad requires more technical skill and operative time than harvesting FPFPs, and usually, the amount of harvested fat become smaller than FPFPs, especially when the damaged lung is far from the pericardium. The amount of harvested FPFPs positively affects air leak prevention;^11^ therefore, longer air leak duration (3.6 ± 3.4 days, *n* = 15) in that study might have been caused by a smaller amount of FPFPs compared with our study.

The risk of loss of the FPFPs in the postoperative period is one of the concerns of the nonsuturing method in this study. In our study, postoperative CT was checked in 13 patients and 10 (76.9%) patients had residual FPFPs in the same position as the fat fixed in the operation. These results suggest the satisfactory preservation of FPFPs in our method.

In conclusion, in this study we have introduced an effective and efficient method for the control of intraoperative air leak using sutureless fixation of FPFPs for lung damage.

## AUTHOR CONTRIBUTIONS

Conception and design: Koike S; Collection and assembly of data: Koike S; Data analysis and interpretation: Koike S; Manuscript writing: Koike S; and final approval of manuscript: all authors.

## FUNDING INFORMATION

None declared.

## CONFLICT OF INTEREST STATEMENT

There are no conflicts of interest to declared.

## Supporting information


**Figure S1.** Type of the damage of lung parenchyma classified. Type 1: intraoperative air leak from staple line (arrow), type 2: delamination of visceral pleura (arrow), type 3: laceration of lung parenchyma (arrow).

**Figure S2.** Flow chart of patients. Between February 2022 and March 2023, 78 patients with lung cancer underwent anatomic lung resection at Japanese Red Cross Society Nagano hospital. A total of 22 patients with type 2 or 3 intraoperative lung parenchymal damage were enrolled in this study. Patients with no lung damage, who had undergone surgery by other operators than S.K. were excluded.

**Table S1.** Time for procedure.

## Data Availability

The data underlying this article cannot be shared publicly for protecting privacy of individuals that participated in this study. The data may be shared on reasonable request to the corresponding author after an additional approval by the Institutional Review Board of Japanese Red Cross Society Nagano hospital, Japan.
