# Supplementary figures and images for: Intraoperative control of air leak using a sutureless free pericardial fat pad covering method in lung cancer resection
Source: Thorac Cancer. 2023 Aug 10;14(25):2627–30. doi: 10.1111/1759-7714.15065 (PMC10481144; doi:10.1111/1759-7714.15065)

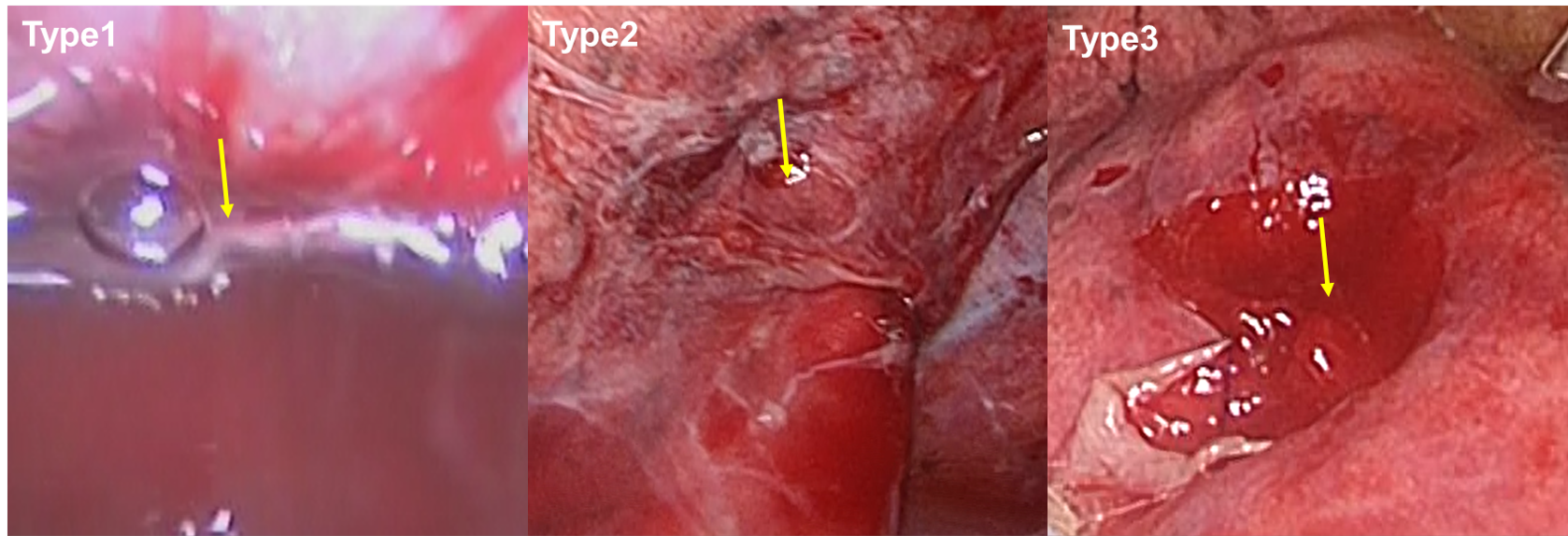

Supplement: Supplementary file 1 — Figure S1. Type of the damage of lung parenchyma classified. Type 1: intraoperative air leak from staple line (arrow), type 2: delamination of visceral pleura (arrow), type 3: laceration of lung parenchyma (arrow). [file TCA-14-2627-s001.tif]

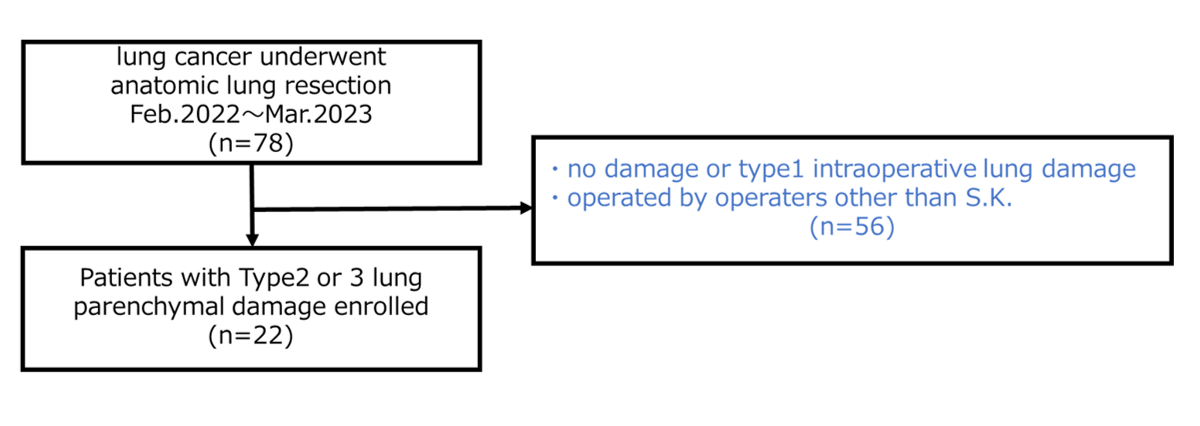

Supplement: Supplementary file 2 — Figure S2. Flow chart of patients. Between February 2022 and March 2023, 78 patients with lung cancer underwent anatomic lung resection at Japanese Red Cross Society Nagano hospital. A total of 22 patients with type 2 or 3 intraoperative lung parenchymal damage were enrolled in this study. Patients with no lung damage, who had undergone surgery by other operators than S.K. were excluded. [file TCA-14-2627-s003.tif]
