# Supplementary material for: Intraoperative control of air leak using a sutureless free pericardial fat pad covering method in lung cancer resection
Source: Thorac Cancer. 2023 Aug 10;14(25):2627–30. doi: 10.1111/1759-7714.15065 (PMC10481144; doi:10.1111/1759-7714.15065)
Supplement: Supplementary file 3 — Table S1. Time for procedure. [file TCA-14-2627-s002.docx]

**Supplemental Table**

| **Table S1. Time for procedure** | | |  |
| --- | --- | --- | --- |
| **FPFP without suture (n=16^a^)** | **FPFP suture^b^ (n=2)** | **lung suture ^c^ (n=5)** |  |
|  |  |  |  |
| 169 (141-262) | 812(693-932) | 421(280-491) |  |
| Data are shown as median (25th, 75th percentiles) ^a^16 of 23 patients checked the time, ^b,c^reference data: Time for FPFP suture and lung suture was  retrospectively investigated. FPFP=free peicardial fat pad | | |  |
|  |  |  |  |
|  |  |  |  |
|  |  |  |  |
